# Progress of GP clusters 2 years after their introduction in Scotland: findings from the Scottish School of Primary Care national GP survey

**DOI:** 10.3399/bjgpopen20X101112

**Published:** 2020-11-18

**Authors:** Stewart Mercer, John Gillies, Bridie Fitzpatrick

**Affiliations:** 1 Professor of Primary Care and Multimorbidity, Usher Institute, University of Edinburgh, Edinburgh, UK; 2 Honorary Professor, Usher Institute, University of Edinburgh, Edinburgh, UK; 3 Honorary Senior Research Fellow, Institute for Health and Wellbeing, University of Glasgow, Glasgow, UK

**Keywords:** general practice, primary health care, managed quality circles, quality improvement, quality of health care

## Abstract

**Background:**

The concept of GP clusters is derived from 'quality circles' in general practice in Europe and Canada. GP clusters commenced across Scotland in 2016 to improve the quality of care of local populations.

**Aim:**

To determine GPs' views on clusters, and the robustness of bespoke questions about them.

**Design & setting:**

A cross-sectional national survey of work satisfaction of GPs in Scotland took place, which was conducted in July 2018–October 2018.

**Method:**

An analysis of bespoke questions on GP clusters was undertaken. The questions were completed by quality leads (QLs) and all other GPs in a nationally representative sample of GPs.

**Results:**

In total, 2456 responses were received from 4371 GPs (56.4%). QLs reported that clusters were meeting regularly, and were friendly and well organised but not always productive. Support for cluster activity (data, health intelligence, analysis, quality improvement methods, advice, leadership, and evaluation) was suboptimal. Factor analysis identified two separate constructs (cluster meetings [CMs] and cluster support [CS]), which were minimally influenced (<2%) by GP and practice characteristics. Non-QLs (75% of all GPs) were generally satisfied with the two-way communication with the cluster QLs, but the great majority (>70%) reported no positive changes in various aspects of quality improvement. Factor analysis of these items indicated two constructs (cluster knowledge and engagement [CKE] and cluster quality improvement [CQI]), which were minimally affected by GP and practice characteristics.

**Conclusion:**

GP clusters are ‘up and running’ in Scotland but are at an early stage in terms of perceived impact and appear to be in need of more support in order to improve quality of care. The bespoke questions developed on clusters have robust construct validity, suitable for future surveys.

## How this fits in

GP clusters were introduced across the whole of Scotland in 2016 as a way of improving integration and quality of care, and are a core part of the new Scottish GP contract. GP clusters or 'quality circles', as they are known in Europe, have been shown to improve various aspects of quality of care in general practice, although Scotland is one of very few countries to introduce them on a compulsory and national scale. The Scottish School of Primary Care conducted a survey of all GPs in Scotland between July 2018–October 2018 (with a 56% response rate), which included bespoke questions on the progress of the clusters. Responses indicated that GP clusters were ‘up and running’ across Scotland but were still at an early stage in terms of perceived impact, and were in need of more support across a range of domains to meet their aims. The bespoke questions developed on clusters had robust construct validity, suitable for future surveys.

## Introduction

GP clusters were introduced nationally in Scotland in April 2016, following the termination of the Quality and Outcomes Framework (QOF) and the initiation of transitional quality arrangements.^1^ In Scotland, clusters are geographical groupings of GP practices, which aim to improve quality of care within and across practices (intrinsic role), and to contribute to health and social care integration (extrinsic role).^2,3^ Each cluster has a GP cluster quality lead (CQL) and each practice has a GP practice quality lead (PQL) who attends the CMs. Clusters are formed by groups of GP practices who choose to work together and are responsible for their own governance via their own professional responsibilities (*‘* *peer-led, values driven* *’*).^1,2^ CQLs, however, are also part of a ‘tripartite’ arrangement with the local NHS health board and the local GP subcommittee of the Area Medical Committee (see Supplementary file for further details). The stated intention of Scottish Government was that QL roles would be functional across Scotland by April 2017,^1^ forming a key part of the evolving new Scottish GP contract.^4^

The concept of GP clusters is derived from quality circles in general practice in Europe and Canada.^5,6^ Quality circles have been shown to improve guideline adherence, prescribing behaviour, and patient safety, although their effectiveness varies considerably.^5,6^ Quality circles or GP clusters have spread rapidly across Europe, with a recent shift in focus from continuing professional development to quality improvement;^7^ however, only a few countries have introduced GP clusters at a national, contractual level. Within the UK, England has recently engaged in GP contract reform, including the establishment of primary care networks, but it will be several years before their impact is known.^8^ Wales, however, introduced GP clusters in 2014 (although unlike Scotland, retained core elements of the QOF).^9^ A report in 2017 by the National Assembly for Wales found wide variation in the pace and scale of change, and made a series of recommendations.^10^ A 2019 report from the Auditor General for Wales, however, stated that: *‘Much work remains to be done to ensure that clusters have a clear remit, broad membership, and are able to drive change at scale and pace*.’^11^ Thus, progress in GP clusters in Wales over the first 5 years appears to have been slow.

The progress of GP clusters in Scotland is unclear, owing to the lack of robust national data.^12^ However, the speed of implementation of health and social care integration in general — of which GP clusters are a part — has been criticised.^13^ In view of this lack of data on progress of GP-cluster working in Scotland, the Scottish School of Primary Care (SSPC) — a consortium of nine universities in Scotland (http://www.sspc.ac.uk) — conducted a survey of all GPs in Scotland in July–October 2018, which included bespoke questions on the progress of clusters. In this article these findings are reported, and the construct validity of the bespoke questions that were included in the survey is also discussed.

## Method

The GP cluster survey was conducted as part of a National GP Worklife Survey on job satisfaction in Scotland (which will be reported separately). It collected demographic information about the GPs and their practice characteristics, plus bespoke questions about the GP clusters, which were developed from a literature review,^5^ and the National Framework for GP Clusters published by the Scottish Government in January 2017.^2^ The latter included the Juran Trilogy^14^ of quality planning, quality improvement, and quality control, and also identified the key support needs of GP clusters: data and health intelligence; tailored facilitation; improvement advice; learning and improvement tools; evaluation and research; and leadership and networking.^2^ The bespoke questions on the GP clusters were reviewed by senior stakeholders in the Scottish Government and other relevant organisations, and by GPs and QLs in one cluster, which resulted in some minor wording changes.

### Cluster variables

QLs (CQLs and PQLs) were asked to what extent CMs were: 1) well organised; 2) friendly; 3) well facilitated; and 4) productive. They answered using a five-point response scale (‘always’, ‘nearly always’, ‘sometimes’, ‘hardly ever’, or ‘never’). QLs were also asked how supported they felt in relation to: 1) data; 2) health intelligence; 3) analysis; 4) quality improvement methods; 5) advice; 6) leadership; and 7) evaluation and research. They responded on a four-point scale (‘fully supported’, ‘almost fully supported’, ‘somewhat supported’, or ‘not at all supported’). Questions also included: the number of hours a month QLs spent in their roles; how often the clusters met; if only GPs attended or other staff too; and whether the focus was mainly on the ‘intrinsic’ role (quality improvement) or the ‘extrinsic role’ (participation in local planning of integrated care) of the clusters.

All GPs were asked about their knowledge and engagement with the cluster as follows: 1) I feel informed about what my cluster is trying to achieve; 2) decisions made by my cluster reflect my views; 3) when I make contact, my PQL is responsive to my queries and concerns; 4) my GP cluster is ‘owned’ by its members and feels like ‘our’ organisation; and 5) I can influence the work of my cluster if I chose to. The GPs rated their answers on a five-point scale from ‘strongly disagree;’ to ‘strongly agree’ (mid-point being ‘neutral’). All GPs were also asked how GP clusters had affected: 1) your understanding of quality planning (how to set quality improvement goals); 2) your understanding of quality improvement (methods and approaches); 3) your understanding of quality control (measuring improvement, ensuring safety); 4) your understanding of the characteristics of your local population of patients (such as age, deprivation, multimorbidity levels); 5) the quality of care you provide; and 6) the extent to which you involve patients in decisions about their care, based on what’s important to them. The answers were measured on a five-point scale from ‘decreased a lot’ to ‘increased a lot’ (mid-point ‘not changed’).

### Participants and representativeness

The survey was sent by post to all 4371 GPs in Scotland between July 2018 and early September 2018, with postal reminders in August and mid-September 2018. Additionally, the survey was sent by email as an online questionnaire in early August, with two further email reminders sent mid and late August. Responses were received from 2465 GPs (56.4%). Representativeness was examined by comparison with national GP data.^15^ The survey was broadly representative in terms of sex, age, rurality, and deprivation (see Supplementary Table S1).

### Statistical analysis

Descriptive statistics were used to characterise the responders and their responses. Factor analysis was performed on the QLs and all the GPs' bespoke cluster questions by means of principle component analysis, with varimax rotation and Kaiser normalisation. The Kaiser-Meyer-Olkin (KMO) measure of sampling adequacy and Bartlett's test of sphericity were calculated. Linear multi-regression analyses assessed the significance and strength of association between independent predictors of mean factor scores. All analysis were done using IBM SPSS Statistics (version 24).

## Results

### GP quality leads' views on clusters

Out of the 2465 GPs who returned a questionnaire, 159 (6.5%) reported being a CQL and 581 (23.6%) a PQL, with 114 (4.6%) being both. Thus, 626 GPs (25.4%) in total had QL roles. Table 1 shows that QLs were slightly older, more often male, but of similar ethnic group to the other GPs. They were more likely to be practice partners, to work <7 sessions a week, and to work in smaller practices than other GPs. The majority voted in favour of the new GP contract in both groups.

**Table 1.** Characteristics of GP quality leads compared with all other GPs

	**GP quality leads, *n* (%)**	**All other GPs, *n* (%)**	***P* value**
**Age, years**			0.001
≤40	135 (23.4)	566 (32.9)	
41–50	219 (37.9)	542 (31.5)	
≥50	225 (38.8)	611 (35.5)	
**Sex**			<0.001
Male	332 (53.2)	687 (37.6)	
Female	292 (46.8)	1138 (62.4)	
**Ethnic group**			0.180
White Caucasian	557 (91.6)	1668 (93.2)	
Other	51 (8.4)	121 (6.8)	
**GP position**			<0.001
Practice partner	589 (94.5)	1453 (80.1)	
Salaried or locum	34 (5.5)	360 (19.9)	
**Sessions per week**			<0.001
<7	192 (30.8)	905 (49.8)	
≥7	431 (69.2)	914 (50.2)	
**Vote in GP Contract**			0.958
Voted in favour	365 (71.9)	922 (72.0)	
Voted against	143 (28.1)	359 (28.0)	
**Practice list size**			<0.001
<5000	250 (40.0)	389 (21.5)	
5000–10 000	289 (46.2)	967 (53.4)	
>10 000	86 (13.8)	454 (25.1)	
**Practice location**			<0.001
Remote and rural	120 (19.3)	225 (12.5)	
Urban	502 (80.7)	1576 (87.5)	
**Deep End practice**			0.839
Yes	72 (25.2)	214 (75.5)	
No	549 (74.8)	1600 (74.5)	

Denominators may vary due to missing data.

**Figure 1.**
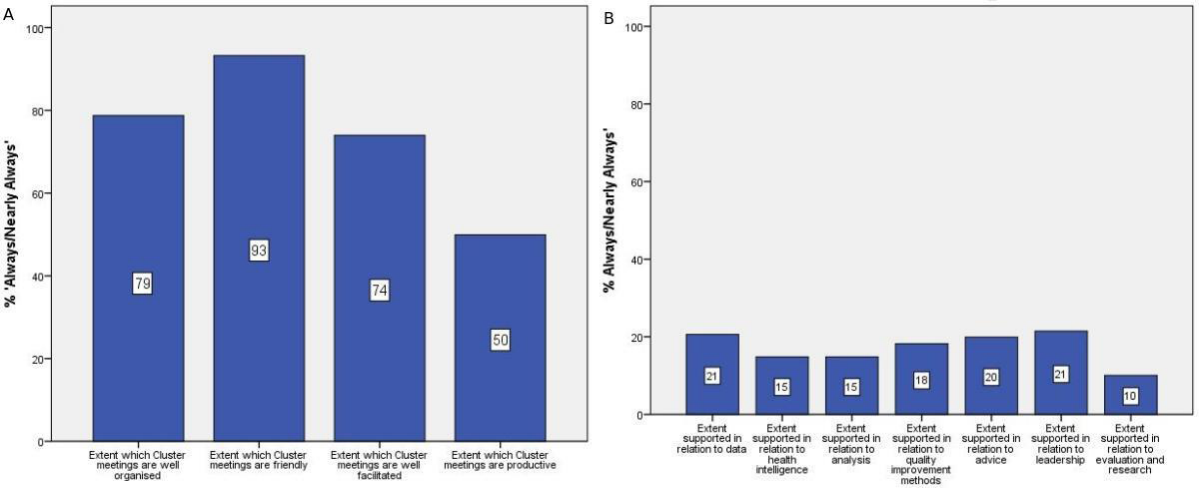
A) GP quality leads' views on cluster meetings; B) GP quality leads' views on level of support for clusters.

Clusters met on average 8.6 (standard deviation [SD] = 3.65; relevant survery responses *n* = 471) times per year. They focused more often on their intrinsic role (65.1% ‘always’ or ‘nearly always’) than their extrinsic role (40.2% ‘always’ or ‘nearly always’). PQLs spent an average of 4.6 hours (SD = 2.93; *n* = 515) a month in the role, and CQLs 8.6 hours (SD = 7.60; *n* = 145) a month. Cluster meetings were generally well organised, friendly, and well facilitated, but not always productive (Table 2 and Figure 1a). QLs mainly felt ‘somewhat supported’ in terms of data, health intelligence, analysis, quality improvement methods, advice, leadership, and evaluation and research (Table 2 and Figure 1b).

**Table 2.** Quality leads' views on cluster meetings and support for cluster activities

	**Always,** *n* (%)	**Nearly always,** *n* (%)	**Sometimes,** *n* (%)	**Hardly ever,** *n* (%)	**Never,** *n* (%)
**Extent to which cluster meetings are**:					
Well organised(*n* = 593)	174 (29.3)	304 (51.3)	84 (14.2)	24 (4.0)	7 (1.2)
Friendly (*n* = 593)	348 (58.7)	218 (36.8)	22 (3.7)	4 (0.7)	1 (0.2)
Well facilitated(*n* = 591)	175 (29.6)	274 (46.4)	110 (18.6)	25 (4.2)	7 (1.2)
Productive (*n* = 593)	88 (14.8)	215 (36.3)	209 (35.2)	65 (11.0)	16 (2.7)
**Extent supported in relation to:**	**Fully supported** *n* (%)	**Almost fully supported** *n* (%)	**Somewhat supported** *n* (%)	**Not at all supported**n(%)	**Not relevant**n(%)
Data (*n =* 595)	31 (5.2)	92 (15.5)	340 (57.1)	117 (19.7)	15 (2.5)
Health intelligence(*n* = 593)	19 (3.2)	69 (11.6)	314 (53.0)	159 (26.8)	32 (5.4)
Analysis (*n =* 594)	15 (2.5)	73 (12.3)	313 (52.7)	172 (29.0)	21 (3.5)
Quality improvement(*n* = 593)	18 (3.0)	91 (15.3)	314 (53.0)	154 (26.0)	16 (2.7)
Advice (*n* = 593)	20 (3.4)	99 (16.7)	317 (53.5)	141 (23.8)	16 (2.7)
Leadership (*n* = 595)	30 (5.0)	99 (16.6)	289 (48.6)	161 (27.1)	16 (2.7)
Evaluation and research(*n* = 594)	7 (1.2)	52 (8.8)	264 (44.4)	222 (37.4)	49 (8.2)

**Table 3.** Factor analysis of GP quality leads' views on clusters

**Cluster item**	**Factor 1^a^**	**Factor 2^a^**
Well organised	0.869	0.140
Friendly	0.829	0.029
Well facilitated	0.883	0.164
Productive	0.816	0.263
Data	0.112	0.823
Health intelligence	0.068	0.852
Analysis	0.078	0.863
Quality improvement	0.138	0.831
Advice	0.232	0.798
Leadership	0.302	0.725
Evaluation and research	0.106	0.787

Bartlett's test of sphericity = *P*<0.001.

KMO measure of sampling adequacy = 0.896.

^a^High factor loadings identify a single construct, which means the items within it can be legitimately aggregated to give a total score.

Factor analysis of the CMs and support items indicated two distinct factors (Table 3, and Supplementary Figure S1). Associations between the mean factor scores and GP age, sex, ethnic group, contract vote, sessions in practice per week, partner or not, practice list size, rurality, deprivation of practice, and whether they were a CQL or PQL was explored by bivariate analysis. CQLs rated the meetings and support significantly higher than the PQLs did but the mean differences were small. None of the other variables were related to the factor scores. Regression analysis (including all the GP and practice characteristics above) confirmed that CQL or PQL was the only independent predictor of both the CM mean score (*P* = 0.004) and CS mean score (*P* = 0.011). However, for both CM and CS scores, the explanatory power of the regression models were very low (*R* ^2^ = 0.016 and 0.014, respectively) indicating that CQL or PQL status had a minimal impact on the variation in mean scores.

### All GPs' views on clusters

In terms of knowledge and engagement, the majority of GPs felt informed about what their cluster was trying to achieve and that their PQL was responsive to queries or concerns (Table 4). However, in terms of clusters and their own quality improvement, the vast majority (<75%) reported no improvement (Table 4). Factor analysis of the bespoke items on cluster knowledge and engagement and quality improvement indicated two distinct factors (Table 5, and Supplementary Figure 2).

**Table 4.** All GPs' views on clusters and quality

	**Strongly disagree,** *n* (%)	**Disagree,** *n* (%)	**Neutral,** *n* (%)	**Agree,** *n* (%)	**Strongly agree,** *n* (%)
I feel informed about what my cluster is trying to achieve(*n* = 2450)	195 (8.0)	463 (18.9)	553 (22.6)	1021 (41.7)	218 (8.9)
Decisions made by my GP cluster reflects my views(*n* = 2447)	147 (6.0)	420 (17.2)	1055 (43.1)	694 (28.4)	131 (5.4)
My practice quality lead is responsive to queries and/or concerns (*n* = 2406)	51 (2.1)	107 (4.4)	843 (35.0)	1063 (44.2)	342 (14.2)
Our GP cluster is owned by its members and feels like our organisation (*n* = 2439)	184 (7.5)	432 (17.7)	1040 (42.6)	615 (25.2)	168 (6.9)
I can influence the work of my GP cluster if I choose to (*n* = 2440)	153 (6.3)	362 (14.8)	884 (36.2)	853 (35.0)	188 (7.7)
**How GP clusters have affected:**	**Decreased a lot,** ***n*** **(%)**	**Decreased a little,** ***n*** **(%)**	**Not changed,** ***n*** **(%)**	**Increased a little,** ***n*** **(%)**	**Increased a lot,** ***n*** **(%)**
Your understanding of quality planning (how to set quality improvement goals) (*n* = 2432)	82 (3.4)	128 (5.3)	1636 (67.3)	530 (21.8)	56 (2.3)
Your understanding of quality improvement (methods and approaches) (*n =* 2430)	73 (3.0)	127 (5.2)	1645 (67.7)	527 (21.7)	58 (2.4)
Your understanding of quality control (measuring improvement, ensuring safety) (*n* = 2430)	75 (3.1)	116 (4.8)	1726 (71.0)	473 (19.5)	40 (1.6)
Your understanding of the characteristics of the local population of patients (*n* = 2431)	56 (2.3)	75 (3.1)	1674 (68.9)	556 (22.9)	70 (2.9)
The quality of care that you provide (*n =* 2431)	38 (1.6)	91 (3.7)	1836 (75.5)	440 (18.1)	26 (1.1)
The extent to which you involve patients in decisions about their care (*n* = 2425)	37 (1.5)	76 (3.1)	1984 (81.8)	297 (12.2)	31 (1.3)

**Table 5.** Factor analysis of all GP views on clusters

**Cluster item**	**Factor 1^a^**	**Factor 2^a^**
Informed about cluster	0.808	0.229
Decisions reflect my views	0.828	0.243
PQL responsive to queries or concerns	0.688	0.157
Feel ‘ownership’ of cluster	0.813	0.251
Can influence cluster work	0.805	0.246
Quality planning	0.299	0.822
Quality improvement	0.270	0.862
Quality control	0.236	0.873
Local population needs	0.292	0.731
Quality of care	0.238	0.718
Shared decision making	0.080	0.707

Bartlett's test of sphericity = *P*<0.001.

KMO measure of sampling adequacy = 0.908.

PQL = practice quality lead.

^a^High factor loadings identify a single construct, which means the items within it can be legitimately aggregated to give a total score.

The CKE factor mean score was associated with age (middle-age group had a higher score than younger or older, *P* = 0.004), practice list size (smaller list had a higher score than larger list, *P* = 0.01), practice employment (partners had a higher score than salaried or other, *P*<0.001), and whether they were a QL or not (CQL or PQL higher than other GPs, *P*<0.001). Regression analysis (including all the GP and practice variables above) indicated that being a QL and being a practice partner were the only two significant independent predictors of CKE score. The explanatory power of the regression model was low (*R* ^2^ = 0.130) and the majority of this was explained by the QL variable (*R* ^2^ = 0. 117).

The CQI mean score was associated with age (lower in those aged ≥50 years, *P* = 0.029), ethnic group (higher in those of non-white ethnic group, *P* = 0.012), contract vote (higher in those who voted in favour, *P*<0.001), partner in the practice (higher in partners, *P*<0.001), deprivation (higher in Deep End practices, *P* = 0.001), list size (higher in smaller list size, *P* = 0.029), and whether a QL (higher, *P*<0.001). Regression analysis identified four independent predictors of CQI score: being a QL (*P*<0.001); voting in favour of the contract (*P*<0.001); working >7 sessions a week (*P* = 0.001); and younger age (*P* = 0.037). However, the explanatory power of the regression model was very low (*R* ^2^ = 0.084) and most of this was explained by the QL variable (*R* ^2^ = 0.062).

The questions that form the four factors (CM, CS, CKE, and CQI) are shown in the Supplementary file.

## Discussion

### Summary

To the authors’ knowledge, this survey is the most comprehensive evaluation of GP clusters in Scotland since their inception in April 2016. The data indicate that GP clusters were, by the latter half of 2018, ‘up and running’ with regular meetings that were friendly, well organised, well facilitated, but not always productive. Support for cluster activity was considered suboptimal by the QLs. Non-QL GPs were generally satisfied with their knowledge and engagement with the clusters, but the great majority (>70%) perceived no positive changes in quality improvement. The bespoke questions developed for QLs and for all GPs had robust construct validity on factor analysis, and were minimally influenced by GP or practice demographics. The finding that CQLs had more positive views on clusters (CM and CS scores) than PQLs is noteworthy, and may relate to their more extensive role (intrinsic and extrinsic) and the fact they have substantially more protected time than PQLs. The finding that the CQLs and PQLs had higher scores than other GPs for the CKE and CQI factor scores is perhaps unsurprising given their roles within the clusters.

### Strengths and limitations

The survey had a high response rate and responders were largely representative of the national GP workforce. The percentage in the survey who voted in favour of the new contract (71.9%) was almost identical to the actual poll (71.5%).^16^ Weaknesses include the lack of free text in the survey, which may have generated useful themes. The cross-sectional nature of the survey means associations cannot imply causality; future longitudinal research is required. All survey data report perceptions; robust measurement of the performance and outcomes of GP clusters are required using other sources of data and information. The survey did not collect information on the process or content of work that the QIs were doing. Further work is required to understand the details of the ‘extrinsic functions’ of the clusters, such as the collaborative roles CQLs are playing with the local health and social care partnerships. Nor did it explore how (or if) PQLs act as ‘change agents’ to improve the quality of care in their practices. Information was also not collected on the range of activities that clusters are carrying out. As far as the authors are aware, there is no published national information on this, although some examples of how local intelligence support team (LIST) analysts have been working with practices to improve ways of working has been published (see Supplementary file).^17^

### Comparison with existing literature

As far as the authors are aware, this is the first published study on the views of GPs on clusters at a national level. A recent (December 2019) small evaluation of CQLs in one health board in Scotland found remarkably similar findings to this present study, with regards to the perceived lack of support.^18^ An unpublished survey of CQLs conducted by Healthcare Improvement Scotland in June 2018 also had similar findings: out of 38 CQLs who completed the survey, 65% agreed or strongly agreed that the cluster had developed a supportive atmosphere and sense of mutual trust, but only 32% felt they had sufficient support from the local Health and Social Care partnership, and only 16% reported having adequate administrative support (S Wilson, personal communication, 2018).

A recent study from Wales reported on the development of a primary care cluster's multidimensional assessment (PCCMA), which had been piloted on 38 CQLs, consisting of 53 indicators across 11 systemic dimensions of primary care.^15^ Reliability and validity analysis was absent, presumably owing to the small sample size. It is noteworthy that the bespoke items in the current study overlap with items and concepts in nine of the 11 PCCMA dimensions.

Studies from Europe and elsewhere on quality circles have tended to be small-scale evaluations with limited generalisability.^6^ However, qualitative studies have helped to elucidate the key characteristics required for quality circles to function effectively.^5,6^ These include the importance of the setting (a friendly and relaxed atmosphere),^19,20^ good facilitation skills,^21^ an understanding of quality improvement,^22^ autonomy to determine what topics and/or clinical areas to address,^23^ and access to relevant data.^5^

### Implications for practice

The Scottish Government has published guidance for GP clusters setting out minimum expected recommendations,^17^ which includes regularity of CMs (every 4–6 weeks), minimum sessions per week for quality leads (two sessions per month for PQLs and four for CQLs) and adequate support for quality improvement, including methodology, data, data intelligence, leadership, and administrative support.

The present study's findings, as in the recent study in Lothian,^18^ and the Healthcare Improvement Scotland survey alluded to above, suggest that although some of these recommendations are being met (for example, the regularity of CMs), many are not. In addition, the fact that GPs as a whole perceive little or no improvements in various aspects of the quality of care they deliver as a result of clusters is concerning. The recent COVID-19 pandemic is likely to have stalled or diverted the progress of the clusters further. Concerted effort and support will be required to increase the pace of development of the clusters post-COVID-19 in order to realise Scotland’s ambitions for GP clusters as a key component of primary care transformation. In practical terms, this will require considerably more support with data, health intelligence, analysis, quality improvement methods, advice, leadership, and evaluation and research for CQLs.
